# Supplementary figures and images for: Mapping the functional form of the trade-off between infection resistance and reproductive fitness under dysregulated immune signaling
Source: PLoS Pathog. 2024 Feb 26;20(2):e1012049. doi: 10.1371/journal.ppat.1012049 (PMC10919860; doi:10.1371/journal.ppat.1012049)

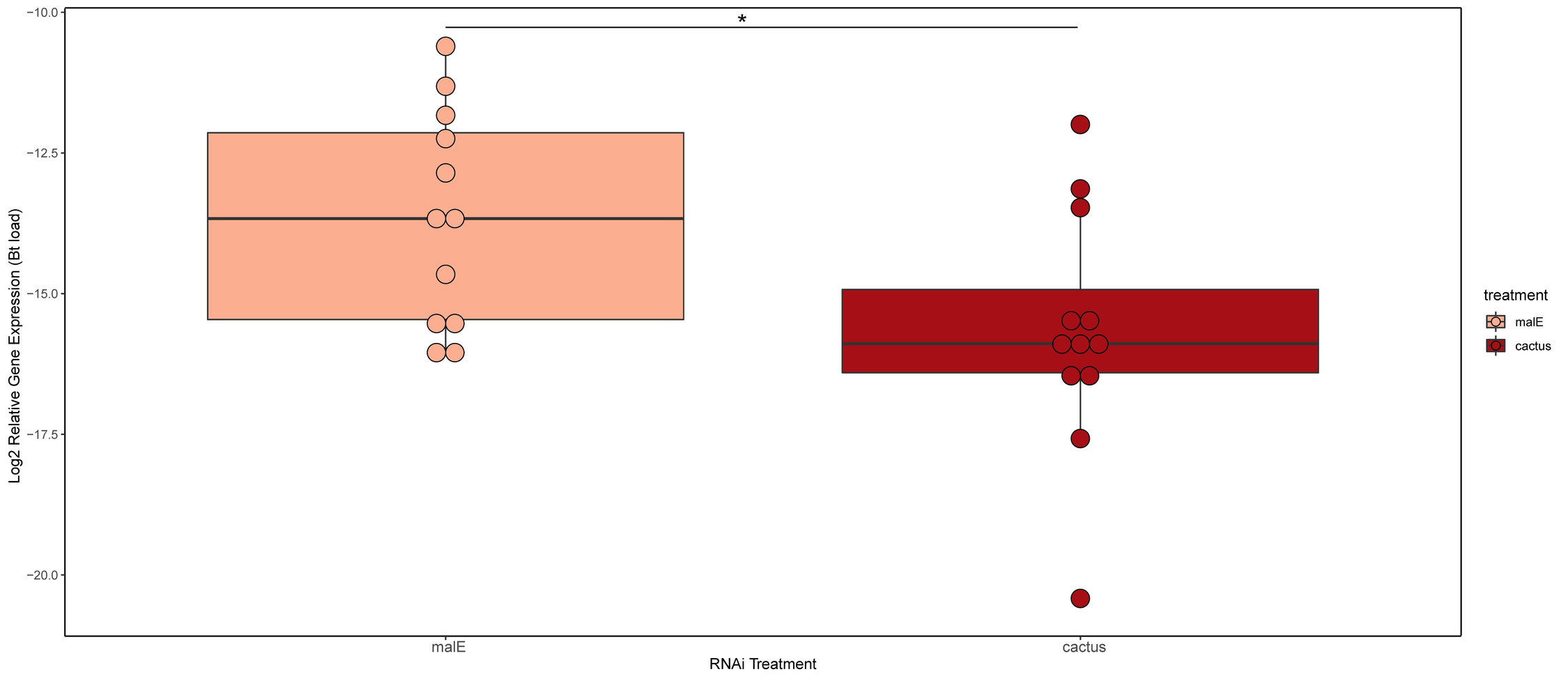

Supplement: S1 Fig — To measure shifts in host resistance to bacterial infection from cactus (250 ng) RNAi treatment, beetles were given an LD-50 dose of Bt and sacrificed six hours later. Relative bacterial density for each individual within each dsRNA treatment was quantified via RT-qPCR and calculated as the difference between Bt-specific and host reference gene expression (RP18s) on a log2 scale. (TIF) [file ppat.1012049.s003.tif]

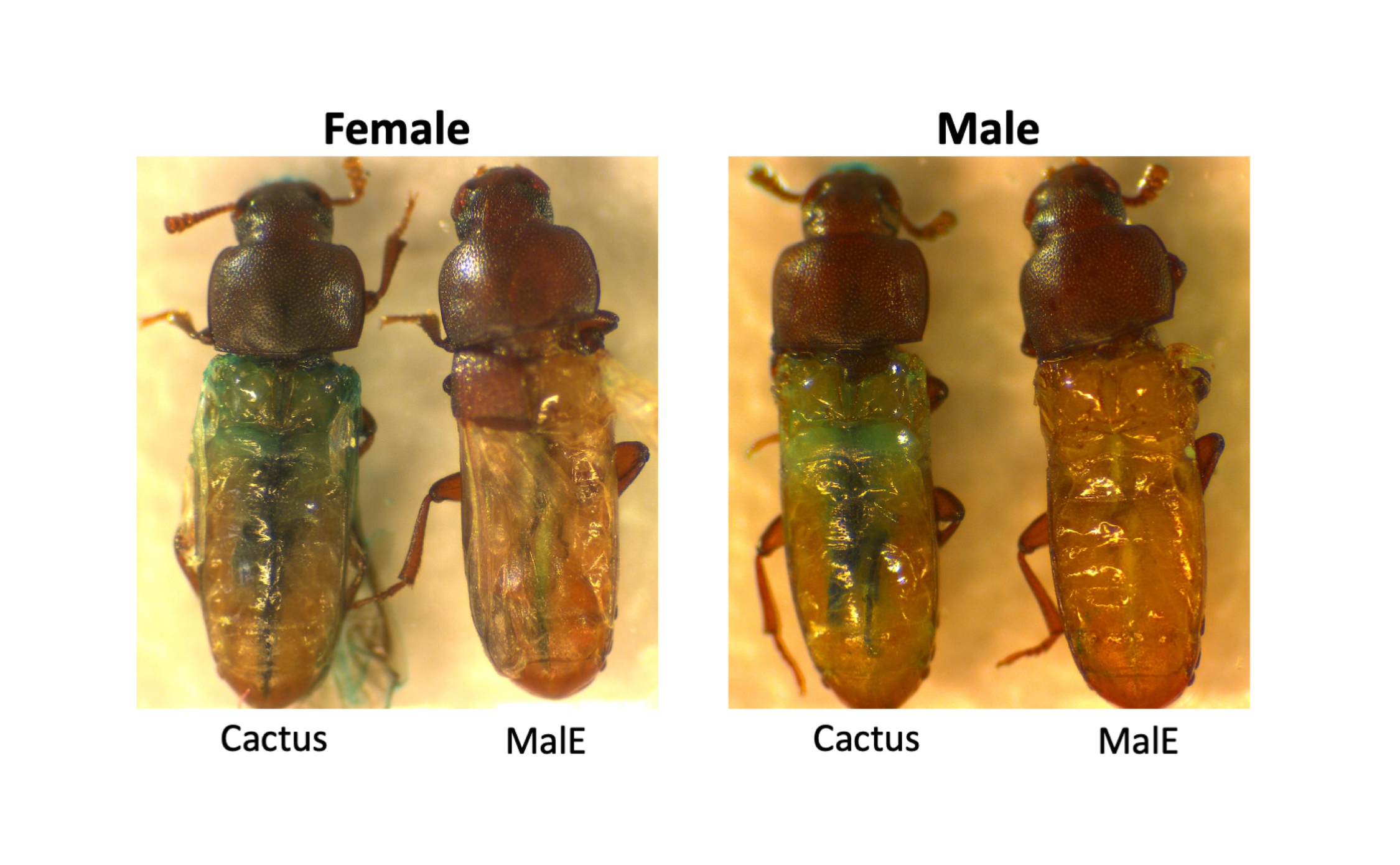

Supplement: S2 Fig — Beetle gut integrity was measured by feeding adults flour stained blue and observing whether the blue dye entered the beetle hemolymph. Beetles with blue food dye in their hemolymph are counted as “smurfs”. (TIF) [file ppat.1012049.s004.tif]

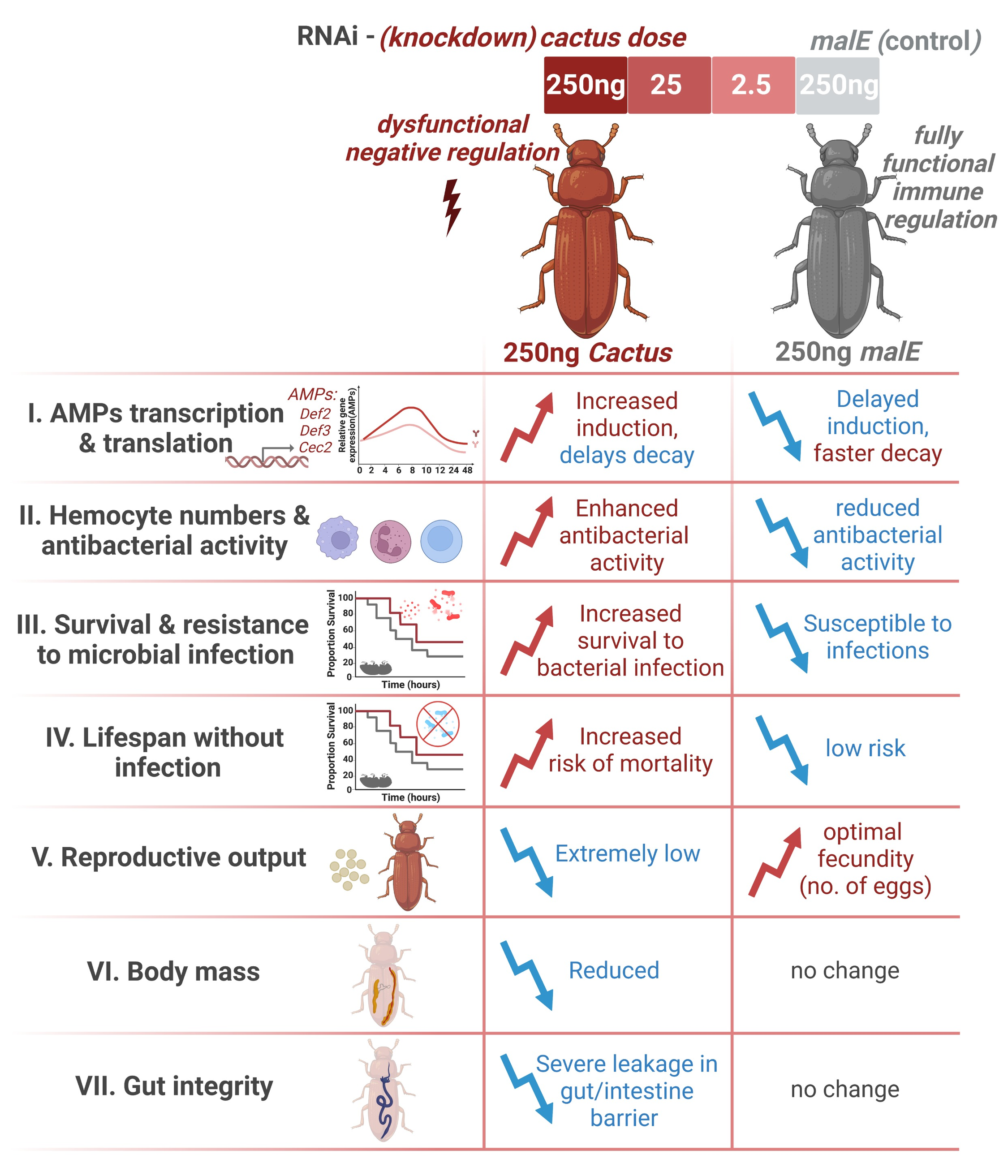

Supplement: S3 Fig — Our study revealed that RNAi-mediated knockdown of cactus, leading to heightened Toll pathway signaling, yields significant increases in (I) AMP transcription, (II) hemocyte numbers, (II) antibacterial activity, and (III) survival against Bt infection. However, this heightened Toll signaling also entails substantial costs, as evidenced by reductions in (IV) beetle lifespan, (V) reproductive output, (VI) body mass, and (VII) gut integrity. Figure created with BioRender.com. (TIF) [file ppat.1012049.s005.tif]

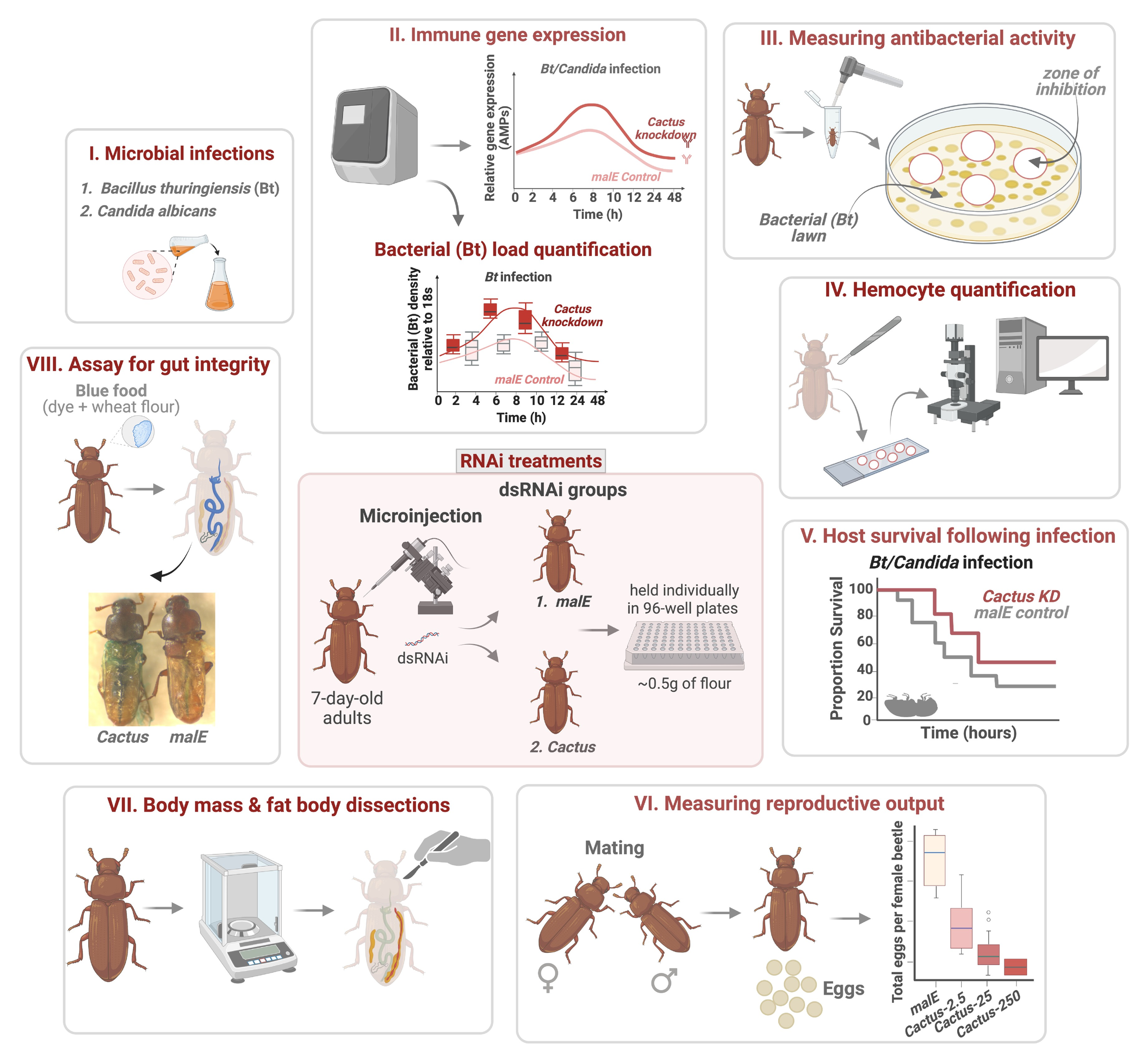

Supplement: S4 Fig — Our study investigated the benefits and costs of enhanced Toll signaling utilizing RNAi with four increasing concentrations of cactus dsRNA. (I) Microbes Bacillus thuringiensis and Candida albicans were used to elicit immune activation. (II) Immune gene expression and Bt load quantification were measured using RT-qPCR. (III) Antibacterial activity was assessed by observing the inhibition of bacterial growth on a lawn of Bt. (IV) Changes in total circulating hemocytes was examined by perfusing the hemolymph in adult beetles, staining, and counting the adhered hemocytes. (V) Survival to pathogen infection was determined by monitoring RNAi treated beetles post-infection with an LD50 dose of Bt. (VI) Female reproductive output was evaluated by pairing treated female beetles with male beetles for 24 hours and counting the number of eggs laid over three days. (VII) Body mass and fat condition were assessed by cleaning and weighing beetles on the 3rd, 4th, and 5th day post-RNAi treatment. Additionally, on the 5th day, beetles were dissected and captured in images for fat body depletion. (VIII) Gut integrity was examined by feeding the beetles media mixed with blue food dye and observing whether the dye entered the hemolymph. Figure created with BioRender.com. (TIF) [file ppat.1012049.s006.tif]

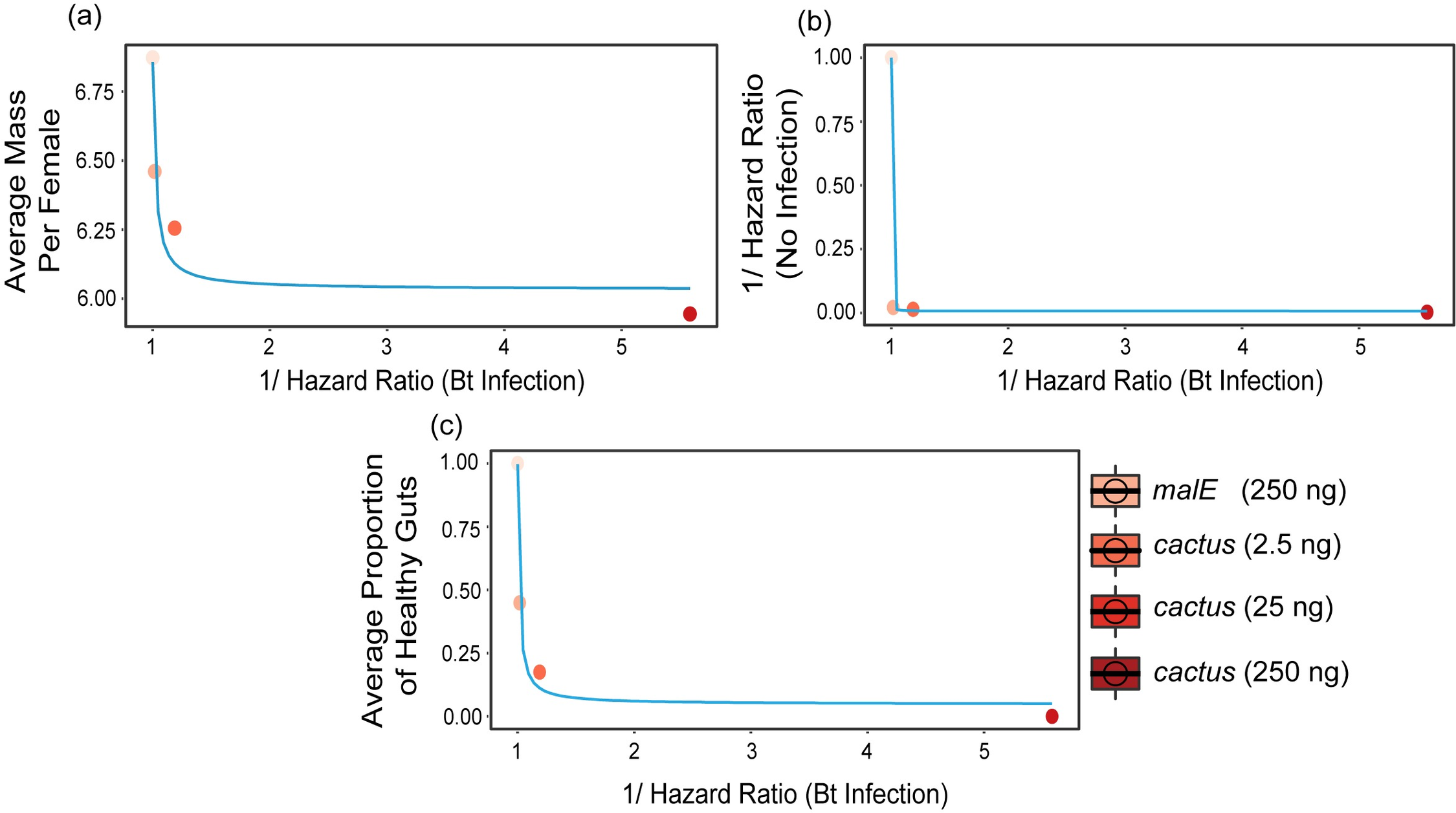

Supplement: S5 Fig — The relationship between infection survival and (a) mass (y = 1 / (-49.2 + 50.4 * x) + 6.0), (b) lifespan (y = 1 / (-3957.3 + 3958.3 * x) + 0.0072, and (c) gut integrity (y = 1 / (-76.5 + 77.6 * x) + 0.048. Survival rate to infection and without infection for each RNAi treatment was calculated as 1/ (the hazard ratio relative to MalE). Average mass per RNAi treatment is the average mass of each treatment across all three days per female. The average proportion of healthy guts is the proportion of beetles without the smurf phenotype across all three days for each treatment. (TIF) [file ppat.1012049.s007.tif]
